# Ship Segmentation in SAR Images by Improved Nonlocal Active Contour Model †

**DOI:** 10.3390/s18124220

**Published:** 2018-12-01

**Authors:** Xiaoqiang Zhang, Boli Xiong, Ganggang Dong, Gangyao Kuang

**Affiliations:** 1 College of Electronic Science, National University of Defense Technology, Changsha 410073, China; bolixiong@gmail.com (B.X.); kgyyeats@163.com (G.K.); 2 National Laboratory of Radar Signal Processing, Xidian University, Xi’an 710000, China; dongganggang@nudt.edu.cn

**Keywords:** synthetic aperture radar (SAR), image segmentation, active contour model (ACM), nonlocal energy

## Abstract

Synthetic aperture radar (SAR) has been widely used in ocean surveillance. As an important part of shipping management and military applications, ship monitoring is a study hotspot in SAR image interpretation; hence, many researches focus on ship targets. Among these studies, ship segmentation is a basic work, but still remains challenging due to the speckle noise and the complicated backscattering phenomenology in SAR images. To solve the problems, this paper proposes a new method for ship segmentation by nonlocal processing. Firstly, the proposed nonlocal energy describes the nonlocal comparison of patches and optimizes regions with spatially-varying features. Secondly, we rewrite the energy functional by introducing a ratio distance defined with respect to the probability density functions of regions to overcome the influence of the multiplicative noise. Finally, the integral histogram is introduced into the pairwise interactions to fasten the speed of convergence. Several rounds of comparative experiments are implemented on real SAR data with different resolutions and bands. The results demonstrate that the proposed method is robust to the speckle noise and intensity variations and could achieve refined segmentation for ship targets.

## 1. Introduction

Due to the capability of working under all weather and all time conditions, synthetic aperture radar (SAR) is an important tool in ocean surveillance. Because of the great significance of ship monitoring in shipping management and military applications, many research works focusing on ship targets have been carried out, such as ship detection, discrimination, and recognition [1,2]. Among these studies, ship segmentation is a fundamental and meaningful work: The accurate segmentation can facilitate subsequent interpretations. On the one hand, segmenting the ship from the background is necessary for extracting target features that are often used in discrimination and classification; on the other hand, the refined segmentation is the pre-processing of the training set in some deep learning-based recognition algorithms, because eliminating the interference of background clutters contributes to classification [3].

**Figure 1:**
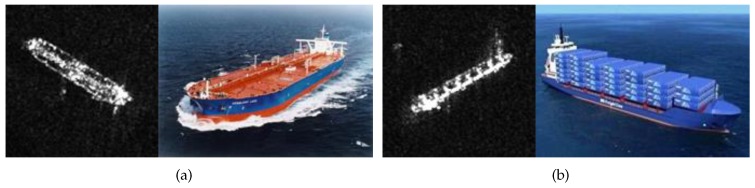
The intensity variations of ships in SAR images. (**a**) Oil tanker; (**b**) carrier.

Though widely studied, object segmentation in SAR images still remains challenging largely due to the two facts. Firstly, the multiplicative speckle noise caused by SAR systems is a common problem. The speckle noise may sharply reduce the image quality and greatly influence image interpretations. Many efforts have been devoted to overcoming the speckle effect. Efficient stochastic models of speckle noise, focusing on the multiplicative nature, have been proposed [4]. Suitable distributions that fit SAR images (e.g., K, Gamma, and $G^{0}$ distributions) are employed to improve the segmentation performance [5,6]. Secondly, the variations of backscattering coefficients also brings much trouble to image segmentation. By contrast, the research in this area is still limited. As is known, the grayscale of a pixel in the SAR image represents the proportion of microwave backscattered from the corresponding region. The intensity values on both sides of the contour are supposed to be homogeneous respectively in many segmentation algorithms. However, this proposition may have some differences in ship segmentation. This is because some specific structures on ships may lead to obvious intensity variations, especially in high resolution SAR images. With the development of the imaging technology, more details can be displayed in images. Therefore, ships appear to have various texture characteristics, instead of bright objects, as shown in Figure 1. Since some dark regions embedded in the ship are similar to the background clutters, it is difficult to extract the ship completely and precisely by the traditional algorithms.

The active contour model (ACM) is a popular tool for image segmentation, due to the ability to locate the object outline accurately. It is a framework for guiding contours toward the boundary of the object by minimizing the energy functional. In some early works, ACMs were mainly based on edge information, such as the snake and the geodesic active contour (GAC) models [7,8]. They utilize edge detectors and guide the contour towards sharp gradients of pixel intensity. However, this kind of ACM is sensitive to noise. To handle the problem, region-based ACMs have been presented, like Chan–Vese (CV), region-scalable fitting (RSF), and local and global intensity fitting (LGIF) models [9,10,11]. They have better noise robustness, since regional information is introduced into the contour evolution. To improve the performance, Wang et al. modified the local region fitting function of the RSF model incorporating a Gaussian distribution and proposed the local Gaussian distribution fitting (LGDF) model [12]. Based on this work, Thieu et al. incorporated the local and global information into a fuzzy energy function and proposed the local and global fuzzy Gaussian distribution (LGFGD) model [13], which achieved success in medical image segmentation. Besides, Some hybrid ACMs were proposed, which absorbed the advantages of those two kinds [14,15]. They have been demonstrated to have good performance in many applications. Variational ACMs have also been widely applied in SAR image segmentation. Most of them are based on the level set due to the high efficiency of contour evolution and the ability to handle the topological change [16].

As an important feature of SAR images, the statistical property is often used to improve the robustness of ACMs. For example, Shuai et al. designed a new energy functional to search for a stationary global minimum, in which the homogeneous regions were modeled by a Gamma distribution [17]. Feng et al. developed a variational multiphase segmentation framework for SAR images, and the $G^{0}$ statistical model was introduced into the regional term of the energy functional to improve the performance [18]. Ayed et al. proposed a level set segmentation method, which measured the conformity of region data to a Weibull distribution representation [19]. Tu et al. combined the modified CV (MCV) model and RSF model by replacing the Euclidean distance by a new ratio distance defined with respect to the probability density functions of regions in SAR images. They integrated the energy functional with Chan’s global minimization active contour framework [20] and proposed global minimization of the modified LGIF (GMLGIF) model [21]. It can be seen that most of these methods are based on the hypothesis that the intensities of targets and the background are homogeneous or follow the same distribution. As we discussed previously, however, this may not be true for ship segmentation in SAR images. That is why the segmentation performance is limited.

In this study, we propose a variational nonlocal ACM for ship segmentation in SAR images. Different from the preceding works, the level set is implemented by the nonlocal comparison of patches. The energy functional consists of two terms: the nonlocal energy term, defined by pairwise interaction, measuring the dissimilarity of patches inside and outside the segmented region, and the regularization term for smooth boundaries. Only the local homogeneity of image features is utilized in the procedure of the nonlocal patch comparison; hence, this method can capture the regions with spatially-varying features. Inspired by Tu’s idea, we introduce the ratio distance into the pairwise interaction and rewrite the nonlocal energy in order to make the method applicable for SAR images. The experiments based on real SAR images show that the proposed method can precisely extract ship targets from the background.

Contributions: This paper proposes an improved segmentation strategy for ship targets in order to overcome the problems of speckle noise and intensity variations. The main contribution of our study can be summarized as follows.

- We introduce the ratio distance, similar to that proposed in [21], into the framework of the nonlocal ACM and apply the improved nonlocal ACM to SAR images. Probability density functions are utilized in nonlocal energy computation, and a prior distribution is not demanded; hence, the proposed model is an unsupervised segmentation method.
- To deal with the large computational cost caused by nonlocal comparisons between patches, we use the integral histogram of a grey image in the patch dissimilarity calculation and accelerate the convergence. In this way, the probability density function of a local patch can be conveniently calculated, with no need to traverse each pixel.
- The influence of parameters τ and σ on segmentation is analyzed. The robust ranges are recommended by means of experiments.

## 2. The Proposed Method

### 2.1. Nonlocal Active Contour Model

Nonlocal image processing, firstly presented by Buades et al., is a scheme dealing with imaging problems using nonlocal comparisons of patches [22]. It has been used in denoising, inverse problems, and classification [23,24]. Jung et al. proposed the nonlocal energy to drive the contour and optimize the homogeneity of the segmented region. It is named nonlocal ACM [25]. Let f:I→R denote the SAR image *f* with the image grid *I* and x,y∈I denote two pixels in the image. A patch around the pixel *x* can be defined as $p_{x}\left(k\right)=f\left(x+k\right)$ with the size (2τ+1)×(2τ+1), where τ is the half patch size. The nonlocal ACM calculates the segmentation by minimizing the following energy functional,

(1) $$E\left(\Omega \right)=E_{\mathrm{NL}}\left(\Omega \right)+\lambda E_{R}\left(\Omega \right)$$

where $E_{R}\left(\Omega \right)$ is a smoothing term regularizing the contour of the region. It is usually defined as the length of the boundary. λ is a weight controlling the contour regularity. More importantly, $E_{\mathrm{NL}}\left(\Omega \right)$ is the nonlocal energy describing the dissimilarity inside and outside region Ω. Let $\Omega^{c}$ denote the complementary region of Ω, then $E_{\mathrm{NL}}\left(\Omega \right)$ is defined as:

(2) $$\begin{array}{c} E_{\mathrm{NL}}\left(\Omega \right)={\bar{E}}_{\mathrm{NL}}\left(\Omega \right)+{\bar{E}}_{\mathrm{NL}}\left(\Omega^{c}\right),\\ {\bar{E}}_{\mathrm{NL}}\left(\Omega \right)=\int_{\Omega \times \Omega }G_{\sigma }\left(x,y\right)d\left(p_{x},p_{y}\right)dxdy \end{array}$$

where the Gaussian kernel $G_{\sigma }\left(x,y\right)$ of scale σ is used as a decaying function of $∥x-y∥$, defined as $G_{\sigma }\left(x,y\right)=e^{-\frac{{∥x-y∥}^{2}}{2\sigma^{2}}}$. $d(p_{x},p_{y})$ plays an important role in this model, evaluating the dissimilarity between patches $p_{x}$ and $p_{y}$. That is, similar patches would lead to a small value of $d(p_{x},p_{y})$; otherwise, $d(p_{x},p_{y})$ should be large. Then, how to measure this kind of patch dissimilarity or distance is the key problem that will be discussed in the next section.

According to the formulas above, it can be seen that the patches in pairwise interaction are not necessarily adjacent, and that is why it is called a nonlocal method. By this means, the nonlocal ACM achieves a good tuning of the scale at which these patches are compared. This is much different from other ACMs.

### 2.2. Patch Comparison in SAR Images

**Figure 2:**
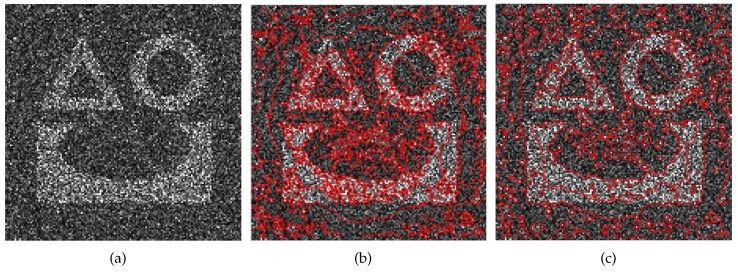
Segmentation by Jung’s algorithms. (**a**) Simulated SAR image; (**b**) segmentation using $L^{2}$ distance; (**c**) segmentation using sliced Wasserstein distance.

As described above, the patch comparison metric $d(p_{x},p_{y})$ has a great influence on the nonlocal energy. To consider the pixel values, the orientation of textures, and the local statistical features jointly, Jung et al. introduced several metrics into comparisons of patches, including $L^{2}$ and Wasserstein distances [25]. Although they have proved to be effective in optical images, they may not be suitable for measuring the distance between SAR image patches due to the speckle noise. To demonstrate this proposition, we present a simple example in Figure 2. A scene of a 256×256-pixel simulated SAR image is segmented by the nonlocal ACM using $L^{2}$ distance and sliced Wasserstein distance, respectively. Speckle noise with a Kdistribution is added to the simulated image in Figure 2a. The experimental results, in (b) and (c), show that these metrics are not applicable to SAR images.

Some related works have been carried out to find an appropriate metric in SAR images. The multiplicative nature of the speckle noise has been investigated by Feng et al., and they proved that the ratio distance is useful to measure the relativity of speckled SAR image patches [18]. According to the research of Deledalle, probability characteristics have satisfactory performance in SAR image processing [26]. Their viewpoints have been further supported by the work of Tu et al. They incorporated the ratio distance into the distribution metric and modified the classic CV model by replacing the Euclidean distance [21]. The energy functional of MCV is defined as:

(3) $$\begin{array}{c} E_{\mathrm{MCV}}\left(\Omega \right)=\lambda_{1}{\bar{E}}_{\mathrm{MCV}}\left(\Omega \right)+\lambda_{1}{\bar{E}}_{\mathrm{MCV}}\left(\Omega^{c}\right)\\ {\bar{E}}_{\mathrm{MCV}}\left(\Omega \right)=\int_{\Omega }{\left(\varepsilon \left\{\log^{2}\left[\frac{P(x,\Omega )}{P_{\mathrm{img}}}\right]\right\}-\varepsilon {\left\{\log\left[\frac{P(x,\Omega )}{P_{\mathrm{img}}}\right]\right\}}^{2}\right)}^{1/2}dx \end{array}$$

where *P* and $P_{\mathrm{img}}$ represent the probability density functions of the region and the whole image, respectively, and ε(·) denotes the mathematical expectation. The robustness to speckle noise of this model has been demonstrated by the experiments based on the MSTARdata [21].

Inspired by all these preceding works, we introduce the distribution metric of the ratio distance into patch comparisons,

(4) $$d\left(x,y\right)={\left(\varepsilon \left\{\log^{2}\left[\frac{P_{x}}{P_{y}}\right]\right\}-\varepsilon {\left\{\log\left[\frac{P_{x}}{P_{y}}\right]\right\}}^{2}\right)}^{1/2}$$

Then, the nonlocal energy can be rewritten as:

(5) $$\begin{array}{c} E_{\mathrm{NL}}\left(\Omega \right)=\int_{\Omega \times \Omega }G_{\sigma }\left(x,y\right){\left(\varepsilon \left\{\log^{2}\left[\frac{P_{x}}{P_{y}}\right]\right\}-\varepsilon {\left\{\log\left[\frac{P_{x}}{P_{y}}\right]\right\}}^{2}\right)}^{1/2}dxdy\\ +\int_{\Omega^{C}\times \Omega^{C}}G_{\sigma }\left(x,y\right){\left(\varepsilon \left\{\log^{2}\left[\frac{P_{x}}{P_{y}}\right]\right\}-\varepsilon {\left\{\log\left[\frac{P_{x}}{P_{y}}\right]\right\}}^{2}\right)}^{1/2}dxdy \end{array}$$

where $P_{x}$ and $P_{y}$ represent the probability density functions of the patches $p_{x}$ and $p_{y}$. The distance can be considered as the standard deviation between the log-likelihood of $P_{x}$ and $P_{y}$. This kind of nonlocal energy is easy to implement, since there is no need for an appropriate statistical model. When the energy functional is minimized, the contour will be driven close to the object boundaries so that the homogeneity of the segmented regions can be optimized.

### 2.3. Acceleration by the Integral Histogram

**Figure 3:**
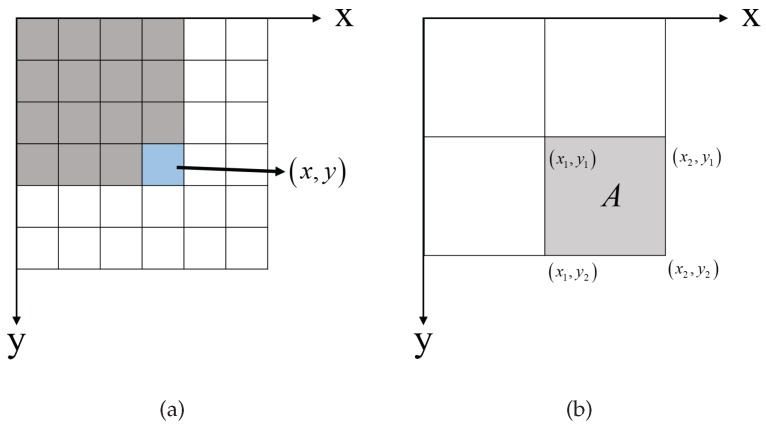
(**a**) Definition of the integral histogram of a grey image; (**b**) fast region computing.

The nonlocal ACM is computationally intensive due to the multiple operations of patch comparisons. Moreover, as shown in (5), the metric used in pairwise interaction requires calculating the probability density functions of each patch. It further increases the computational cost. This disadvantage leads to the low speed of contour evolution in nonlocal ACM. Since the probability density function of a patch can be calculated by the histogram of this region, the integral histogram is introduced into the nonlocal processing to overcome this drawback. It is a useful tool to accelerate the feature computing [27]. In Figure 3a, the integral histogram of a grey image *f* can be defined as follows:

(6) $$IH\left(x,y\right)=IH\left(x-1,y\right)+IH\left(x,y-1\right)-IH\left(x-1,y-1\right)+O\left(f\left(x,y\right)\right)$$

where the integral histogram value $IH\left(x,y\right)$ denotes the histogram of the top-left region of position $\left(x,y\right)$ and $O\left(f\left(x,y\right)\right)$ represents the histogram operation for the intensity $f\left(x,y\right)$. The integral histogram is helpful for the fast region computation. As shown in Figure 3b, the histogram of a rectangle *A* can be quickly obtained by the integral histogram values of the four vertexes.

(7) $$H\left(A\right)=IH\left(x_{2},y_{2}\right)-IH\left(x_{1},y_{2}\right)-IH\left(x_{2},y_{1}\right)+IH\left(x_{1},y_{1}\right)$$

### 2.4. Numerical Implementation

For numerical implementation, the contour of the target can be evolved by a level set function φ. Then, the nonlocal energy can be rewritten as:

(8) $$\begin{array}{c} E_{\mathrm{NL}}\left(\varphi \right)=\int_{I\times I}\left[1-\left|H(\varphi (x))-H(\varphi (y))\right|\right]G_{\sigma }\left(x,y\right)d\left(p_{x},p_{y}\right)dxdy \end{array}$$

where H(·) is the Heaviside function. The regularization term can be defined as:

(9) $$\begin{array}{c} E_{R}\left(\varphi \right)=\int_{I}\left|\nabla H(\varphi (x))\right|dx \end{array}$$

According to the gradient descent algorithm in [25], we minimize the energy and implement the segmentation. The evolution of the level set can be described with with an artificial time t≥0 as follows,

(10) $$\frac{\partial \varphi }{\partial t}=-\left(\nabla E\left(\varphi \right)\right)=-\left(\nabla E_{\mathrm{NL}}\left(\varphi \right)\right)+\lambda \left(\nabla E_{R}\left(\varphi \right)\right)$$

Then, we discretize the gradient flow by the gradient descent as follows:

(11) $$\varphi^{\left(i+1\right)}=\varphi^{\left(i\right)}-\xi \left(\nabla E_{\mathrm{NL}}\left(\varphi^{\left(i\right)}\right)+\lambda \nabla E_{R}\left(\varphi^{\left(i\right)}\right)\right)$$

where ξ>0 is the time step size. The evolution stops when $\left|E^{\left(i+1\right)}-E^{\left(i+1\right)}\right|<\omega$, where ω>0 is a user-defined threshold.

The main steps of the proposed method are summarized as follows:

**Input**: A SAR image *f*, threshold ω, parameters τ, σ and λ, initialization $\varphi_{0}$.

**Output**: The segmentation result $f_{s}$.

1.    Initialization: i←0, $\varphi^{\left(0\right)}\leftarrow \varphi_{0}$.
2.    **Repeat** until $\left|E^{\left(i+1\right)}-E^{\left(i+1\right)}\right|<\omega$:
3.    Computing the integral histogram of the image *f*.
4.    Computing the probability density functions of the image and each patch by the integral histogram.
5.    Computing the patch dissimilarities by the distribution metric of the ratio distance.
6.    Computing the energy gradient, according to Equations (8)–(10).
7.    Update $\varphi^{\left(i+1\right)}$ with $\varphi^{\left(i\right)}$, according to (11).
8.    i←i+1
9.    **End**
10.    $f_{s}\leftarrow H\left(\varphi \right)$

### 2.5. Computational Complexity

Suppose the SAR image size is W×H. In the beginning, computing the integral histogram of the SAR image is independent of other iterative operations, and its computational complexity is in the order of W·H. Then, since the probability density functions of patches can be conveniently calculated by the integral histogram using (7), the computational complexity of patch comparisons is also in the order of W·H. Therefore, the computational complexity of the proposed method is O(W·H).

**Figure 4:**
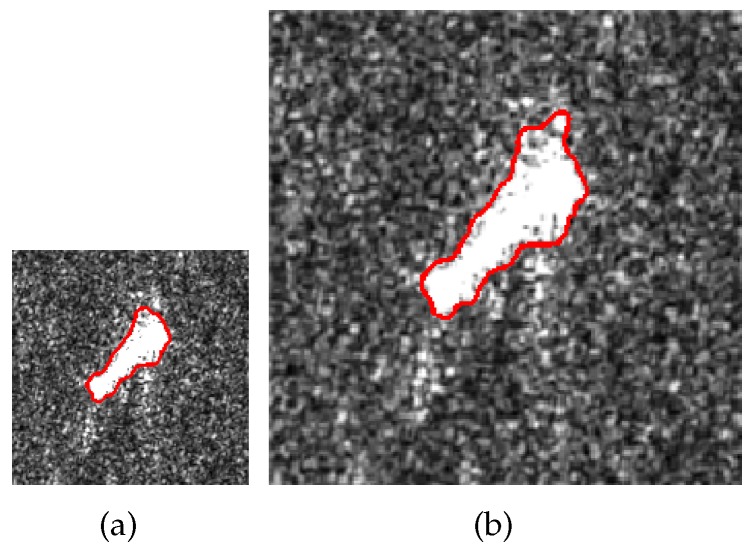
Segmentations of images (**a**,**b**) with sizes of 103×103 and 206×206.

We expand the size of an image from 103×103 to 206×206 and implement the proposed method on the original and enlarged images, respectively. The segmentation results are shown in Figure 4. The running times of (a) and (b) are 16.79 s and 73.52 s. Notice that the enlarged image is four-times the size of the original one, and the ratio of the running time of (b) to that of (a) is approximately four. Therefore, the derivation of the complexity is reliable.

## 3. Experimental Results and Analysis

In this section, We first compare the proposed method with other previous works based on real SAR images and demonstrate the acceleration of convergence by the integral histogram. Then, several ship chips are used to analyze the segmentation accuracy in detail. Finally, we discuss the parameters of the proposed method.

### 3.1. Segmentation of SAR Images

**Figure 5:**
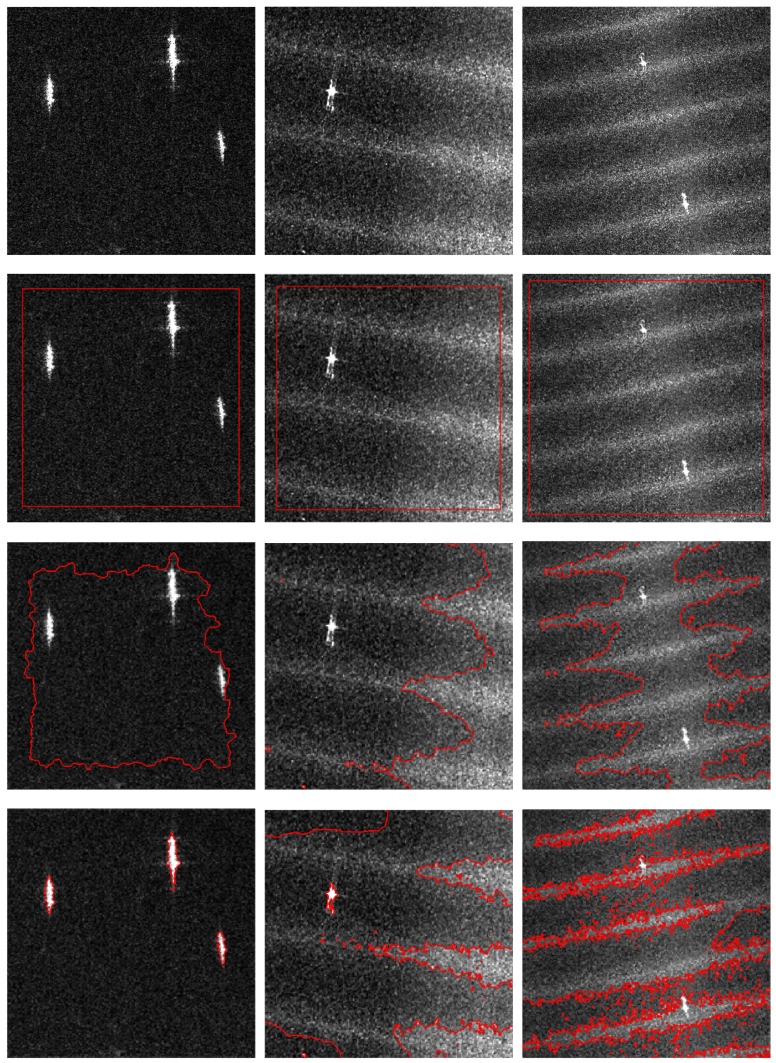

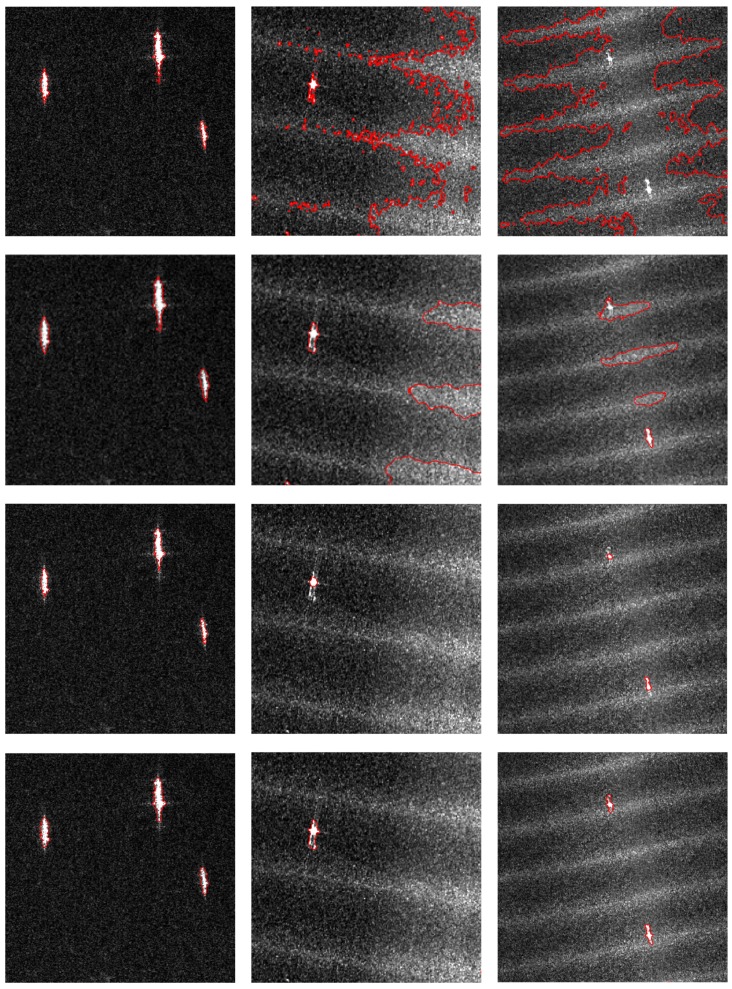
Segmentation of SAR images. The top row shows the real SAR images for the segmentation experiment. From left to right: Subimage 1 is from an X-band Cosmo-Skymed image with 3-m resolution; Subimage 2 and Subimage 3 are from two C-band RADARSAT-2 images with 5-m resolution. The second row shows the initial contours. The third to the seventh rows show the segmentation results of Chan–Vese (CV), region-scalable fitting (RSF), local and global intensity fitting (LGIF), local and global fuzzy Gaussian distribution (LGFGD), and global minimization LGIF (GMLGIF), respectively. The bottom row shows the the segmentation results of the proposed method.

Three SAR images of ocean scenes were used in this experiment. The first one was a subimage (Subimage 1) from an X-band Cosmo-Skymed image with 3-m resolution, and the other two (Subimage 2 and Subimage 3) were from two C-band RADARSAT-2 images with 5-m resolution. Their sizes were 310×310, 400×400, and 660×660. We carried out the proposed method with the parameters τ=3,σ=15,λ=15 and drew a comparison with the CV, RSF, LGIF, LGFGD, and GMLGIF models based on the three images. The original images and the experimental results are shown in Figure 5. It can be seen that the CV model failed to detect the ships in the three images. It was sensitive to the speckle noise and the intensity variations in the background, so the contour evolution was very influenced. The segmentation was not finished after 1000 iterations. As for the other methods, all of them detected the ships and achieved satisfactory results in Subimage 1, because the ships and the background were respectively homogeneous. In Subimages 2 and 3, however, the serious noise and local intensity variations of the background severely interfered with the segmentations of RSF and LGIF. LGFGD did not show good performance either. This may be because the Gaussian distribution was not able to characterize the statistical behavior of the sea clutter in SAR images [5,28]. GMLGIF and the proposed method, with due consideration of the speckle noise, were more suitable for SAR images and overcame those interferences. However, the segmentation by GMLGIF was not precise, and it only extracted the bright region of the ship. By contrast, the segmentation accuracy of the proposed method was higher.

We compare the running time of the convergence by RSF, LGIF, LGFGD, and GMLGIF models and the proposed method in Table 1. The experiments were carried out with MATLAB code running on the same hardware platform of an AMD Athlon (tm) Dual-Core 3.1-GHz CPU and 8.0 GB memory. It can be seen that the original patch comparison was time consuming, but the nonlocal ACM became much faster with the help of the integral histogram.

### 3.2. Segmentation of Target Chips

**Figure 6:**
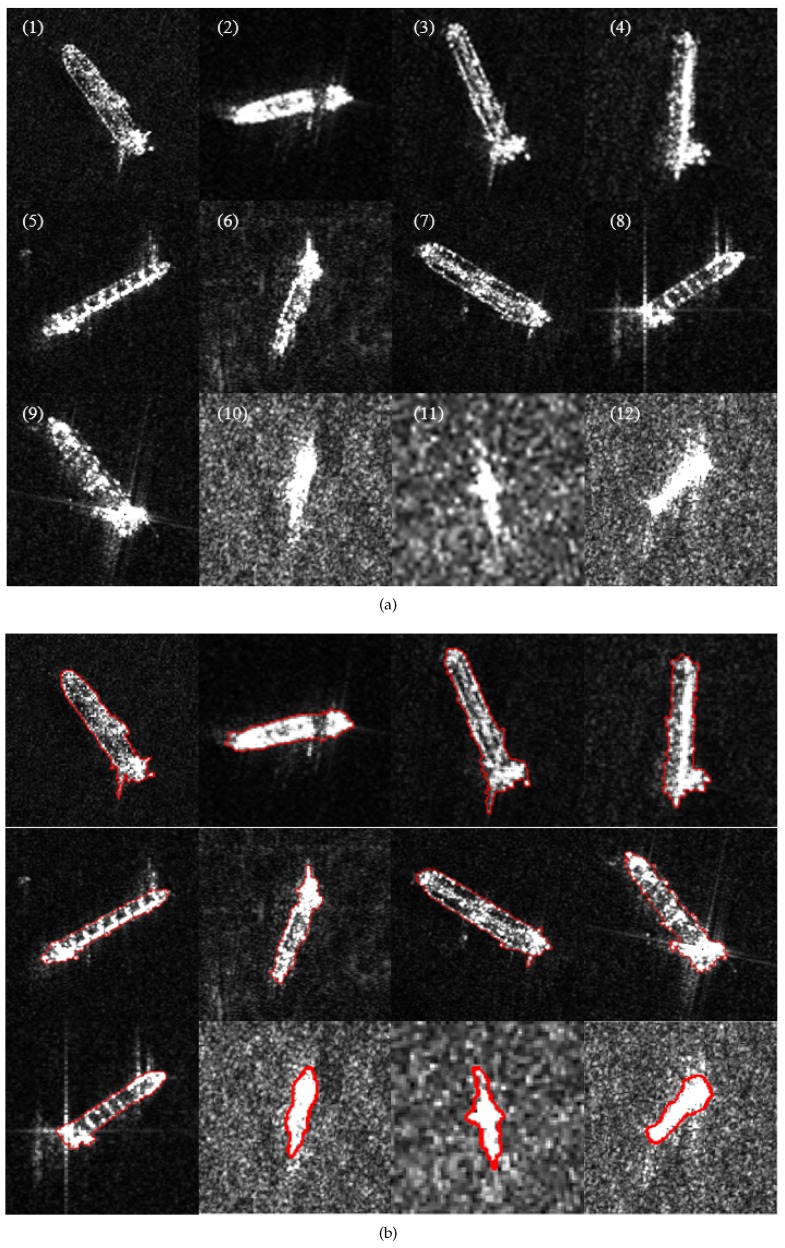

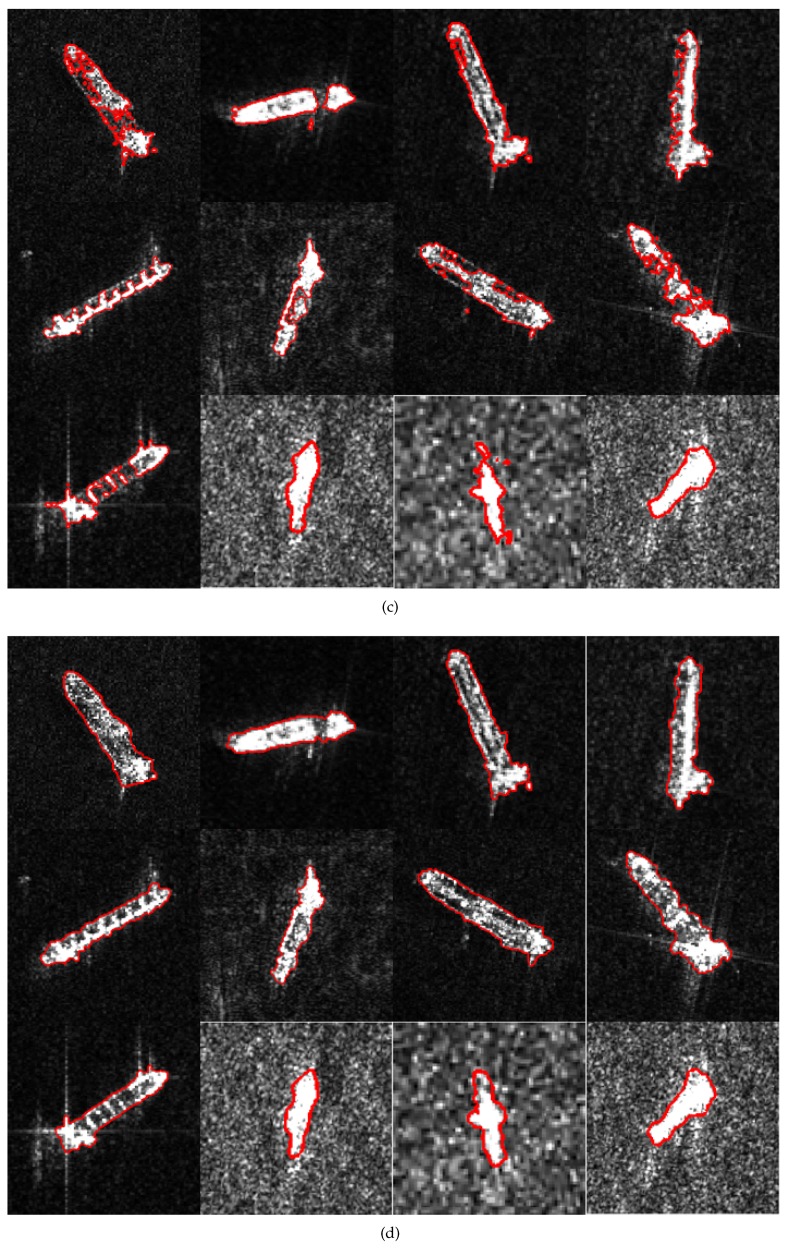
Segmentation of target chips. (**a**) Original chips; (**b**) manual segmentation for reference; (**c**) segmentation by the GMLGIF model; (**d**) segmentation by the proposed method.

Since both the GMLGIF model and the proposed method utilize the modified ratio distance, it is necessary to make a further comparison of these two methods in order to demonstrate the advantage of the nonlocal ACM. Figure 6a shows the 12 chips of the ship target used in this experiment. Chips 1–9, with sizes of 243×243–295×295, were from TerraSAR X-band images with 1-m resolution. Chips 10–12, with sizes of 75×75–103×103, were from RADARSAT-2 C-band images with 5-m resolution. As references, the manual segmentations are shown in Figure 6b. The results of the GMLGIF model are shown in Figure 6c. The proposed method was carried out in Figure 6d, with τ=3,σ=15,λ=15. In low resolution SAR images, a ship target may appear to be a group of bright pixels, like Chips 10–12. Both methods achieved satisfactory results when dealing with Chips 10 and 12. In Chip 11, the contours of the GMLGIF model fell into local minima due to the strong speckle noise. High resolution SAR images showed more structure information of targets; hence, ships appeared to have some texture characteristics, like Chips 1–9. The intensity variations were usually caused by the complicated structure of the deck erection. Under the circumstances, the GMLGIF model failed to deal with the intensity variations on ships. Some dark regions could be be adjusted by regularization. As a result, some ships extracted by GMLGIF were incomplete, while some were segmented into isolated regions. By contrast, the ship contours obtained by the proposed method were closer to the references.

**Figure 7:**
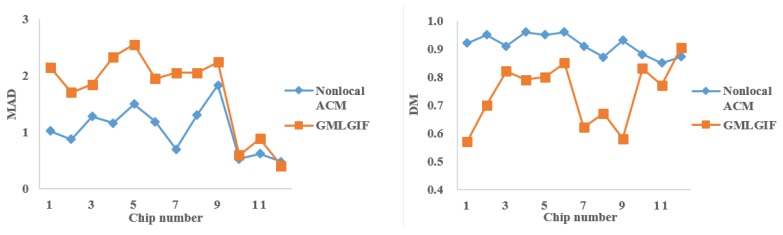
Quantitative evaluation. The mean absolute distances (MADs) of segmentation are on the left, and the Dice metrics (DMs) of segmentation are on the right.

The segmentation performance was quantitatively evaluated by the mean absolute distance (MAD) [29] and the Dice metric (DM) [30]. If there are *n* points on the snake contour $S=\left\{s_{1},s_{2},\ldots ,s_{n}\right\}$, and *k* points on the manually-segmented contour $M=\left\{m_{1},m_{2},\ldots ,m_{k}\right\}$, MAD can be defined as:

(12) $$\mathrm{MAD}(S,M)=\frac{1}{2}\left(\frac{1}{n}\sum_{i=1}^{n}d(s_{i},M)+\frac{1}{k}\sum_{j=1}^{k}d(m_{j},S)\right)$$

where $d\left(s_{i},M\right)=\min_{j}∥s_{i},m_{j}∥$ is the distance from $s_{i}$ to the closest point of M. DM is a measure of contour overlap, which is given by:

(13) $$\mathrm{DM}=\frac{2A_{\mathrm{sm}}}{A_{s}+A_{m}},\mathrm{DM}\in \left[0,1\right]$$

where $A_{s},A_{m},A_{\mathrm{sm}}$ are the segmented contour area, the manually-segmented area, and their intersection area, respectively. A higher DM indicates a better match between two contours. In Figure 7, the quantitative evaluation demonstrates that the contours extracted by the proposed method were more precise.

### 3.3. Discussion of Parameters

The proposed method contains some parameters, such as τ, σ, and λ, which were empirically set in the given experiments. λ is the weight adjusting the regularity of the region boundary, often used in many other ACMs. In this section, we analyze the influence of the other two parameters, related to the patch comparison of the nonlocal processing.

**Figure 8:**
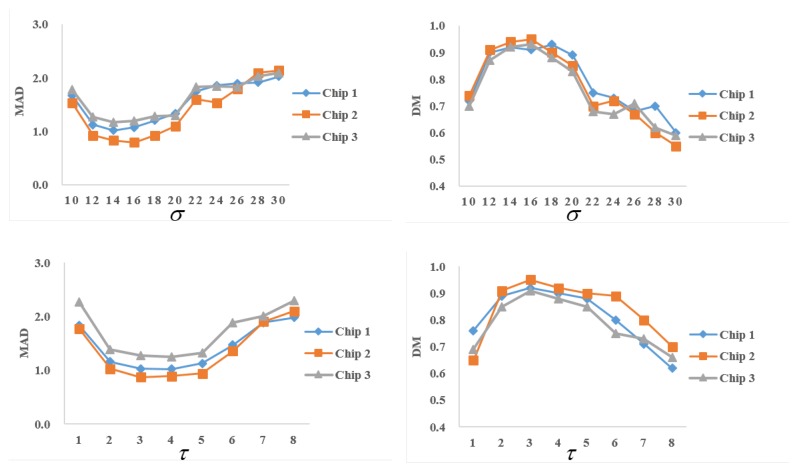
Parameter analysis. Top row: MADs and DMs vary with σ by fixing τ=3. Bottom row: MADs and DMs vary with τ by fixing σ=15.

Considering that τ is the half patch size and σ is the scale of the Gaussian kernel, we suppose that these two parameters are independent from each other. Therefore, the control variate method was adopted in parameter analysis. In other words, we changed one parameter and fixed the other one at a time. The experiments were implemented on the first three chips in Figure 5. Firstly, we set τ = 3 and the range of σ from 10–30. The corresponding MADs and DMs of the segmentation results with various values of σ are shown in the top row of Figure 8. It can be seen that σ, in the range from 12–20, was appropriate for these images. When σ was beyond this range, the segmentation performance degraded. σ is the scale of the Gaussian kernel, and it controls the weight of patch comparison according to the Euclidean distance between patches. Too small of a σ would greatly weaken the influence of the interaction between nonadjacent patches. As a result, the model cannot reach the global optimal segmentation. However, if σ becomes too large, the interaction between patches of different categories would lead to segmentation error. Generally speaking, an image made of an almost constant pattern demands a large σ. By contrast, fast feature variations requires a small one.

Secondly, we fixed σ = 15 and range of τ from 1–10 to test the influence of τ on segmentation. The quantitative evaluations are shown in the bottom row of Figure 8. The half patch size τ directly influences the local statistical description of a pixel. If a patch is too small, it is hard to estimate its local statistics. Otherwise, too big of a patch is no longer locally homogeneous, and it reduces the segmentation accuracy. When τ was set from 2–5, the segmentation kept relatively robust.

## 4. Conclusions

Aiming at the problems of the multiplicative noise and variations of backscattering coefficients, we proposed an improved nonlocal ACM for ship segmentation in SAR images. A new nonlocal energy, incorporating the distribution metric of a ratio distance, was defined to optimize regions with spatially-varying features. The integral histogram was utilized in pairwise interactions to reduce the computational cost. The advantages of the proposed method were verified by several rounds of experiments on the real SAR images from different sensors. The results demonstrated that the proposed method was able to segment ship targets from speckled SAR images with intensity variations. Compared with some existing algorithms, the proposed method achieved obvious improvement in refined segmentation.

## Figures and Tables

**Table 1:** Comparison of the running time. RSF, region-scalable fitting.

	RSF [10]	LGIF [11]	LGFGD [13]	GMLGIF [21]	Nonlocal ACM with Integral Histogram	Nonlocal ACM without Integral Histogram
Subimage 1	104.39 s	110.14 s	85.71 s	87.56 s	82.38 s	331.71 s
Subimage 2	126.79 s	130.16 s	101.03 s	96.76 s	103.60 s	398.23 s
Subimage 3	233.77 s	241.75 s	248.91 s	278.76 s	260.60 s	1167.23 s
